# Rapid Analysis of Deoxynivalenol in Durum Wheat by FT-NIR Spectroscopy

**DOI:** 10.3390/toxins6113129

**Published:** 2014-11-06

**Authors:** Annalisa De Girolamo, Salvatore Cervellieri, Angelo Visconti, Michelangelo Pascale

**Affiliations:** Institute of Sciences of Food Production, National Research Council of Italy (ISPA-CNR), Via G. Amendola 122/O, 70126 Bari, Italy; E-Mails: salvatore.cervellieri@ispa.cnr.it (S.C.); angelo.visconti@ispa.cnr.it (A.V.); michelangelo.pascale@ispa.cnr.it (M.P.)

**Keywords:** deoxynivalenol, FT-NIR, rapid method, wheat, LDA, PLS

## Abstract

Fourier-transform-near infrared (FT-NIR) spectroscopy has been used to develop quantitative and classification models for the prediction of deoxynivalenol (DON) levels in durum wheat samples. Partial least-squares (PLS) regression analysis was used to determine DON in wheat samples in the range of <50–16,000 µg/kg DON. The model displayed a large root mean square error of prediction value (1,977 µg/kg) as compared to the EU maximum limit for DON in unprocessed durum wheat (*i.e.*, 1,750 µg/kg), thus making the PLS approach unsuitable for quantitative prediction of DON in durum wheat. Linear discriminant analysis (LDA) was successfully used to differentiate wheat samples based on their DON content. A first approach used LDA to group wheat samples into three classes: A (DON ≤ 1,000 µg/kg), B (1,000 < DON ≤ 2,500 µg/kg), and C (DON > 2,500 µg/kg) (LDA I). A second approach was used to discriminate highly contaminated wheat samples based on three different cut-off limits, namely 1,000 (LDA II), 1,200 (LDA III) and 1,400 µg/kg DON (LDA IV). The overall classification and false compliant rates for the three models were 75%–90% and 3%–7%, respectively, with model LDA IV using a cut-off of 1,400 µg/kg fulfilling the requirement of the European official guidelines for screening methods. These findings confirmed the suitability of FT-NIR to screen a large number of wheat samples for DON contamination and to verify the compliance with EU regulation.

## 1. Introduction

Deoxynivalenol (DON), also known as vomitoxin, is a type B trichothecene mycotoxin. It is one of the major secondary metabolites produced by fungi of the *Fusarium* genus, mainly *Fusarium graminearum* and *Fusarium culmorum*, and occurs predominantly in grains, such as wheat, maize, barley, oats and rye [1]. DON inhibits the synthesis of DNA, RNA and proteins and has a hemolytic effect on erythrocytes. DON can cause feed refusal, vomiting, reduced weight gain, diarrhea, hemorrhage, skin lesions, growth depression and immunosuppression, which have a negative impact on human and animal health [2,3,4,5,6]. In order to protect consumers from exposure to DON through the consumption of cereal-based food products, the European Commission has set maximum permitted levels for DON ranging from 200 µg/kg for processed cereal-based food for infants and young children up to 1,750 µg/kg for unprocessed durum wheat, maize and oats [7].

Analytical methods for rapid, sensitive and accurate determination of DON in foods and feeds are highly demanded for exposure risk assessment studies and to enforce regulatory requirements issued by governments and international organizations. A number of analytical methods, such as gas chromatography (GC) with electron-capture or mass spectrometric (MS) detection and high-performance liquid chromatography (HPLC) based on UV or MS detection have been developed to quantitatively measure DON concentration in cereals and derived products [8,9,10]. Although these traditional methods are sensitive and accurate, most of them involve expensive and time-consuming steps, including sample cleanup and detection, being unsuitable for screening purposes. Large amounts of cereals are processed in the food and feed industry each year, and frequent checks are required to verify the compliance of raw materials with regulation, resulting in a large number of samples to be analyzed. Factors, like promptness and low cost of analysis, minimal sample preparation and environmentally-friendly methods, are of paramount importance to rapidly respond to the demands of the market. In the last few decades, a variety of rapid methods based on competitive enzyme-linked immunosorbent assays (ELISA) or on novel technologies, including lateral flow devices (LFD), membrane-based flow-through enzyme immunoassay, fluorescence polarization (FP) immunoassay, molecularly imprinted polymers (MIP) and surface plasmon resonance (SPR) biosensors, have been reported for the rapid analysis of DON [8,11,12]. However, these methods are destructive, require an extraction step and, in some cases, a clean-up procedure. Recently, a rapid, easy to-perform and non-invasive method using an electronic nose based on metal oxide sensors to distinguish the quality of durum wheat samples based on the content of DON has been reported [13]. Infrared spectroscopy (IR) has gained wide acceptance within food and feed analysis as a rapid analytical tool that requires minimal or no sample preparation, and in contrast with traditional chromatographic analysis, it does not require reagents, nor does it produce chemical waste. Near-infrared (NIR) or mid-infrared (MIR) spectroscopy techniques, both in combination or not with Fourier-transform (FT), are commonly used in a remarkably wide range of applications for the analysis of moisture, oil, fiber, starch, lipids, protein, yeast and bacteria in agricultural products [14,15]. In recent years, the potential of using IR spectroscopy for the detection of mycotoxins, including DON, ochratoxin A, fumonisins and aflatoxins, and mycotoxigenic fungal contamination in cereals and cereal products has been also demonstrated [16,17,18,19,20,21,22,23,24,25,26,27,28,29,30,31,32,33,34,35,36,37]. Among mycotoxins, the most investigated one was DON, mainly in *Fusarium*-damaged wheat kernels and ground wheat [16,18,19,20,22,23,24,34] and to a lesser extent in maize, barley and oat [17,21,32,33]. The majority of these IR methods for the determination of DON in wheat is based on NIR or UV-Vis-NIR spectroscopy. The feasibility of using FT-NIR spectroscopy for the qualitative and quantitative prediction of DON in unprocessed ground durum and common wheat was reported for the first time by our research group [19]. Performance results suggested the use of FT-NIR as a sorting tool for screening purposes; however, both qualitative and quantitative models merited further implementation in a larger study involving more wheat samples with a homogeneous distribution of DON levels around the EU maximum limit (*i.e.*, 1,750 µg/kg) for unprocessed durum wheat in order to make the model more robust and reliable [19]. Moreover, the classification model included both common and durum wheat samples and used a cut-off level (300 µg/kg) far from the EU maximum limit (ML) to distinguish the two classes of wheat samples [19]. FT-NIR spectroscopy for DON determination in whole wheat kernels was also reported by some authors [23,34]. The advantages of FT include improvement in the spectral reproducibility and the accuracy and precision of wavelength discrimination [28].

The objective of this research was to develop a robust, rapid and inexpensive FT-NIR method for the analysis of DON-contaminated ground durum wheat samples and to verify the compliance with European regulations. Both quantitative and classification models were developed and validated to establish the most suitable approach to estimate DON in unprocessed durum wheat. The proposed FT-NIR classification method as a first screening step for DON detection in samples could provide a high-throughput analysis platform that could improve food and feed safety at mills and could be used for monitoring programs.

## 2. Results and Discussion

DON levels in the 464 durum wheat samples varied from <50 µg/kg (quantification limit of the HPLC method) to 16,000 µg/kg, with mean and median values of 2,390 and 1,100 µg/kg, respectively. This concentration range covered the majority of DON concentrations found in the routine surveillance samples in wheat supply chains and was appropriate for the scope of the study to develop calibration and classification models. Although the positive skewness and kurtosis values of DON concentrations indicated that the number of highly-contaminated wheat samples was less than that of wheat samples with no or low values of DON contamination, there was an approximately fifty-fifty distribution of DON levels around the EU ML for unprocessed durum wheat (1,750 µg/kg); in particular, 57% of the tested samples contained DON levels less than ML, whereas the remaining 43% of samples exceed this threshold.

**Figure 1:**
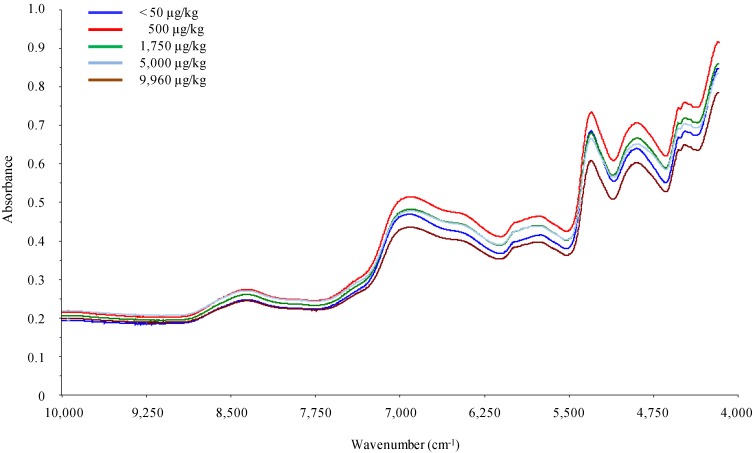
FT-NIR spectra of five different ground unprocessed durum wheat samples contaminated with increasing levels (from <50 to 9,960 µg/kg) of deoxynivalenol (DON) (as measured by the reference HPLC method).

Wheat samples were analyzed by FT-NIR spectroscopy, and spectra were recorded as absorbance between 10,000–4,000 cm^−1^. Figure 1 shows the FT-NIR raw spectra of five different unprocessed ground wheat samples contaminated in the range of <50 µg/kg to about 10,000 µg/kg DON. From the comparison of these spectra, it appears that wheat samples contaminated with low DON levels have FT-NIR bands in common with those containing high DON levels, thus indicating that the major functional groups and chemical constituents co-exist in both types of samples.

### 2.1. Quantification of DON in Contaminated Wheat Sample

**Figure 2:**
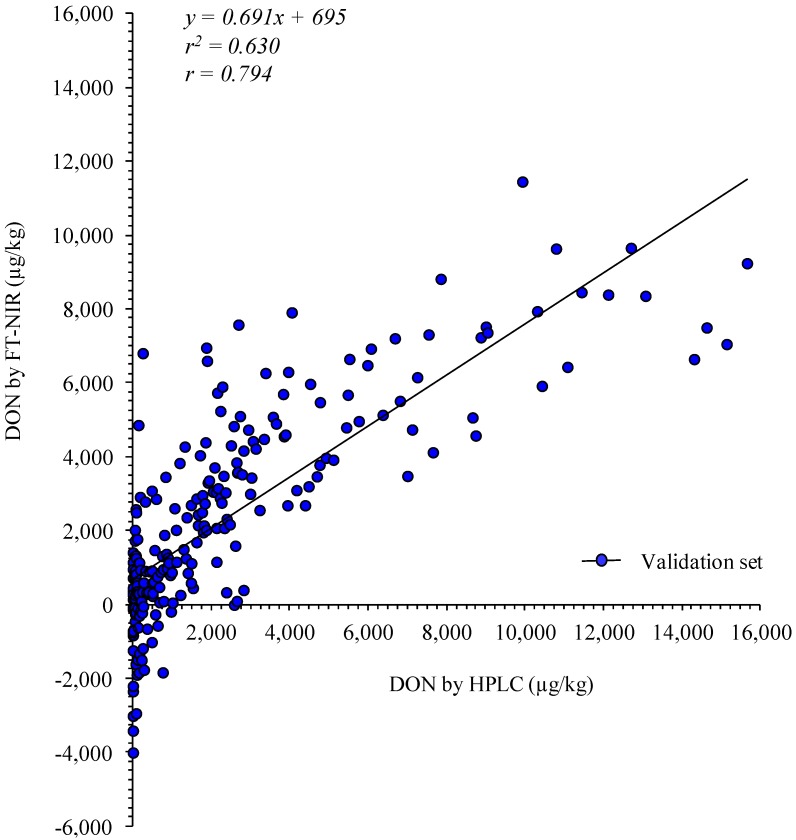
Partial least squares (PLS) regression plot of measured (by HPLC) and estimated (by FT-NIR) DON concentrations in the validation set (model PLS I).

In our previous work, we evaluated the ability of using FT-NIR spectroscopy for the determination of DON in durum wheat at levels between <50 µg/kg and 2,700 µg/kg by using the statistical approach of partial least squares (PLS) regression [19]. The performance results provided good evidence of the feasibility of using FT-NIR spectroscopy as a screening tool for the determination of DON in wheat samples. However, the inhomogeneous distribution of DON levels around the EU ML for unprocessed durum wheat (*i.e.*, 94% of samples with DON levels <1,750 µg/kg) made the model poorly robust [19]. Based on these findings, we further implemented the PLS model in a larger study involving more calibration and validation samples with a larger concentration range of DON. The resulting PLS model (PLS I) covered the range of DON concentration from <50 to 16,000 µg/kg and included 232 samples for the calibration set and 232 for the validation one. The model was developed with eight PLS factors, explaining 80% of the total variance of the entire set of calibration data. The slope and the coefficient of determination (*r^2^*) values (both 0.802) of the calibration regression curve indicated that the PLS model can be used for screening and approximate calibrations with a root mean squares error of calibration (RMSEC) value of 1,473 µg/kg DON. However, the *r^2^* value of the validation regression curve was 0.630, indicating that the PLS model was only usable for a rough screening of wheat samples and showed a root mean squared error of prediction (RMSEP) of 1,977 µg/kg DON that was considered very large with respect to the EU ML. Furthermore, based on the residual predictive deviation (RPD) (1.72) and range error ratio (RER) (6.89) values, the model had a very poor classification ability and was not recommended for any purpose. Figure 2 shows the PLS validation plot of the measured data (by HPLC) in relation to the estimated data (by FT-NIR).

It is well known that fungal infections of kernels cause multiple changes of kernel composition and pigmentation, thus causing spectral variability [38]. To evaluate if high contamination levels of DON were responsible for the poor classification ability of PLS I, samples containing DON levels between 6,000 and 16,000 µg/kg DON were excluded from the dataset, and another PLS model was developed (PLS II). The model included 204 samples for the calibration set and 204 for the validation one with 65% of wheat samples with DON levels less than 1,750 µg/kg. With respect to PLS I, similar results were obtained for PLS II in terms of slope (0.749) and intercept (427 µg/kg), whereas the values of RMSEC and RMSEP were 753 µg/kg and 868 µg/kg, respectively. Although the RMSEP value of model PLS II was lower than that obtained with model PLS I, in both cases, it corresponded to approximately 14% of the entire range of DON concentration in the samples used in the models. As for model PLS I, the RPD value (1.66) indicated that model PLS II was not recommended for any purpose.

PLS results obtained in the present study were in agreement with those reported by Dvoracek *et al.* [23], which applied the FT-NIR spectroscopy to the determination of DON in wheat kernels in the range of 0–13,000 µg/kg and 0–5,000 µg/kg DON. Based on these observations we concluded that the PLS approach was unsuitable for the aim of the study; therefore, the classification one was used.

### 2.2. Classification of DON Contaminated Wheat Samples

The classification LDA models proposed herein have been developed on a huge number of durum wheat samples with a broad range of DON contamination levels. The resulting models covered the range of DON concentration from <50 to 16,000 µg/kg and included 232 samples for the calibration set and 232 for the validation one. Initially, the spectra were treated using principal components analysis (PCA). The first 10 principal components, accounting for more than 99% of the total variance, were selected as input variables for the LDA. Two different approaches were used to develop LDA models in order to find the most suitable one to estimate DON in durum wheat samples. With the first approach (LDA I), wheat samples were classified into three groups based on DON contamination levels: Class A (DON content less than 1,000 µg/kg), Class B (DON content ranging from 1,000 to 2,500 µg/kg) and Class C (DON content more than 2,500 µg/kg). A discriminant model was then developed to classify ground wheat samples into the three DON contamination groups. The overall classification rate was 82% during the calibration process. In particular, 77% of acceptable samples were correctly classified into Class A; 75% of wheat samples with DON levels to be confirmed with a reference method were correctly classified as Class B; and 94% of rejectable samples were correctly classified into Class C. The model was then validated by using an independent dataset. Results are reported in Table 1.

**Table 1:** Validation results of the linear discriminant classification model (LDA I). The first column indicates the class (A, B and C) assigned by the HPLC reference analysis, whereas the other three columns refer to the class predicted by LDA analysis.

Assigned class ^a^ (by HPLC reference analysis)	Number of samples classified in the predicted classes (by FT-NIR analysis)		
	A	B	C
A	92	22	4
B	6	18	13
C	2	12	63
Overall classification rate (%)		75	
FC samples (%) ^b^		3	
FNC samples (%) ^c^		7	

^a^ A: DON ≤ 1,000 µg/kg; B: 1,000 µg/kg < DON ≤ 2,500 µg/kg; C: DON > 2,500 µg/kg; ^b^ FC, false compliant; ^c^ FNC, false, not compliant.

**Figure 3:**
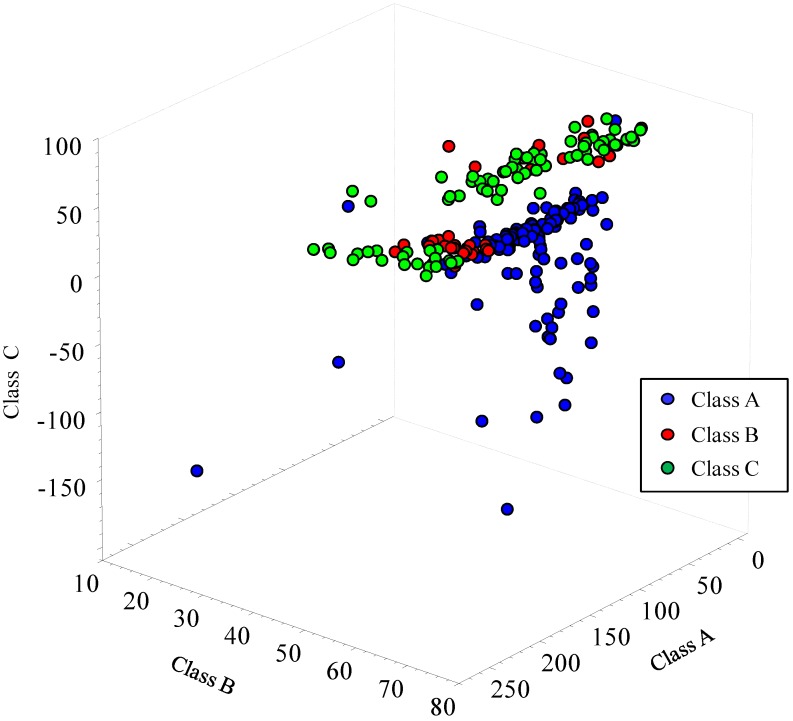
Linear discriminant analysis score plot for FT-NIR spectra of wheat samples naturally contaminated with varying DON content (validation set). Class A: DON ≤ 1,000 µg/kg; Class B: 1,000 µg/kg < DON ≤ 2,500 µg/kg; Class C: DON > 2,500 µg/kg.

The validated model achieved an overall classification rate of 75% with a comparable classification rate for Class A and Class C (approximately 80%) and lower for Class B (49%). In particular, wheat samples belonging to Class B were classified as either Class A (16%) or Class C (35%) (Table 1). From Figure 3, showing the LDA score plot of wheat samples naturally contaminated with DON and classified into three contamination groups, a scattered distribution of samples of Class A and a partial overlapping of Class B to the contiguous Classes A and C is clearly evident. By looking inside these overlapped samples, five of 22 samples of Class A that were incorrectly classified as Class B had DON levels close to the cut-off of 1,000 µg/kg (*i.e.*, from 700 to 1,000 µg/kg). Similarly, nine of 13 samples of Class B that were incorrectly classified as Class C were contaminated with DON levels close to the cut-off of 2,500 µg/kg (*i.e.*, from 1,900 to 2,500 µg/kg). The low discrimination ability of the model around the cut-off limits was probably due to the lower number of samples representing Class B as compared to the other two classes in the calibration set, thus making the model poorly balanced for wheat samples with contamination levels in the range of 1,000–2,500 µg/kg DON. The European official guidelines for analytical methods recommend that only those validated methods that have a false compliant (FC) rate <5% at the level of interest shall be used for screening purposes, whereas suspected non-compliant results shall be confirmed by a confirmatory method [38]. Despite the low discrimination ability of the model LDA I for samples belonging to Class B, the amount of FC samples (eight out of 232) accounted for 3% and fulfilled the requirement of the European official guidelines for screening methods, making the proposed model suitable for screening purposes.

With the second approach, wheat samples were classified into two groups based on a cut-off level of DON to distinguish them. Three different LDA models were developed using cut-off levels of 1,000, 1,200 and 1,400 µg/kg DON (LDA II–IV, respectively), in order to find the optimal threshold allowing the highest classification rate of wheat samples. The overall classification rate in the calibration processes was in the range of 91%–92% with FC values of 3%–7%. When the models were validated, the overall classification rates were still good (89%–90%, Table 2) and in agreement with those obtained in the calibration process, indicating the good robustness of the developed LDA models. Moreover, model LDA IV using a cut-off of 1,400 µg/kg fulfilled the requirement of the European official guidelines for analytical methods [38] and was considered the most reliable one for the screening of DON in unprocessed durum wheat. Good results in terms of repeatability (<2%) and within-laboratory reproducibility (<3%) were also obtained for all LDA models, indicating the robustness of the measurements and the instrumental stability over a period of time.

Considering that wheat samples used in the calibration and validation sets belonged to different cultivars or mixtures of cultivars and were obtained in different periods from field crops located in different Italian regions, it was unrealistic to expect 100% correct classification. Although a slight misclassification occurred, advantages in terms of costs and the rapidity of analysis makes classification model LDA IV a useful screening tool for the analysis of a large number of durum wheat samples and to verify the compliance with legislation by reducing the number of samples to be confirmed with a reference method.

**Table 2:** Validation results of the linear discriminant classification models (LDA II-IV) using different cut-off levels of DON.

Classification results	Discrimination models (cut-off DON, µg/kg)		
	LDA II (1,000)	LDA III (1,200)	LDA IV (1,400)
Overall classification rate (%)	89	90	90
FC samples (%) ^a^	7	6	5
FNC samples (%) ^b^	4	4	5

^a^ FC, false compliant; ^b^ FNC, false, not compliant.

Classification results obtained in the present paper represent a great improvement in terms of the robustness of the model and DON correct prediction as compared to those reported in our previous work [19]. These findings are also better than those reported by Dvoracek *et al.* [23], which applied the LDA to classify 400 intact wheat grain samples into two classes using four different cut-off levels in the range of 1,250 to 30,000 µg/kg DON. Although performance indexes were 89%–93%, the number of misclassified samples increased with the decreasing of the cut-off level, with the model using a cut-off of 1,250 µg/kg DON giving approximately 40% of misclassified samples. These results were probably related to the use of intact wheat kernels, which made the use of FT-NIR spectroscopy perform poorly at contamination levels close to the EU ML [23]. On the other hand, our findings indicate that the grinding step of unprocessed wheat is a useful step that helps to overcome the inhomogeneity problem and allows one to obtain a representative sample to be analyzed by FT-NIR spectroscopy.

## 3. Experimental Section

### 3.1. Chemicals

Acetonitrile of HPLC grade was purchased from Mallinckrodt Baker (Milan, Italy). Ultrapure water was produced by a Millipore Milli-Q system (Millipore, Bedford, MA, USA). Deoxynivalenol (DON) standard was purchased from Sigma-Aldrich (Milan, Italy). DON immunoaffinity columns (DONtest™ HPLC) were obtained from Vicam, a Waters Business (Milford, MA, USA), glass microfiber filters (Whatman GF/A) and paper filters (Whatman No. 4) from Whatman (Maidstone, UK). The PTFE syringe filters with a diameter of 25 mm and a pore size of 0.45 mm were bought from Teknokroma (Barcelona, Spain).

### 3.2. Durum Wheat Samples

A total of 500 samples (1 kg each) of naturally contaminated durum wheat of several different cultivars or mixtures of cultivars was obtained from field crops located in different Italian regions during the period 2008–2013. After manual homogenization, an aliquot of each wheat sample (about 200 g) was finely ground by the Tecator Cyclotec 1093 (International PBI, Hoganas, Sweden) laboratory mill equipped with a 500-µm sieve and analyzed by FT-NIR spectroscopy and then by HPLC. An additional laboratory quality control (QC) sample of unprocessed durum wheat naturally contaminated with 1,420 ± 39 µg/kg DON was used to evaluate the repeatability and intra-laboratory reproducibility of the best prediction model.

### 3.3. HPLC Analysis

DON quantitative determination was performed according to the method described by Visconti *et al.* [39], with minor modifications. Briefly, 25 g of ground sample was extracted with 100 mL of phosphate buffer solution (PBS, 10 mM sodium phosphate, 0.85% sodium chloride, pH = 7.4) by blending at high speed for 2 min with a Sorvall Omnimixer (Sorvall Instruments, Norwalk, CN, USA). The extract was filtered through both filter paper (Whatman No. 4) and glass microfiber filter (Whatman GF/A), and 2 mL of the filtered extract were loaded onto the DONtest™ immunoaffinity column. After washing the column by passing 5 mL of water through it, DON was recovered by eluting 1.5 mL of methanol. The eluate was dried under an air stream at 50 °C, re-dissolved into 250 µL of the mobile phase (acetonitrile/water, 8:92; *v*/*v*), and 50 µL were injected onto the HPLC system (Agilent 1100 Series, Agilent Technology, Palo Alto, CA, USA) with the diode array detector set at 220 nm.

### 3.4. FT-NIR Analysis

FT-NIR spectra were recorded using an Antaris II FT-NIR spectrophotometer (Thermo Electron Corporation, Madison, WI, USA) equipped with an interferometer, an integrating sphere working in diffuse reflection and an indium and gallium arsenide (InGaAs) detector. A sample-cup spinner allowed the automatic collection of several sub-scans from each sample that were averaged to obtain representative spectra. The integrating sphere’s internal reference was also used to collect the background spectrum. Approximately 30 g of ground wheat samples were placed on the rotary sample-cup spinner, and 64 interferometer sub-scans in ranges from 10,000 to 4,000 cm^−1^, with a resolution of 8 cm^−1^, were applied for the collection of each spectrum sample by means of the software, Results Integration v3.0.197 (Thermo Electron Corporation, Madison, WI, USA, 2006).

### 3.5. Spectral Data Preprocessing and Outlier Identification

The final spectral data were imported as SPA/SPG (Omnic) format into The Unscrambler^®^ X, v10.1 (CAMO Software AS, Oslo, Norway, 2011) software in order to perform multivariate statistical analysis. Some preliminary descriptive analyses were performed by both graphic tools (histogram or spectra visualization) and numerical results (mean, minimum, maximum, standard deviation, number of missing data, *etc.*). Then, to remove the spectral baseline shift, noise and light scatter influence, some spectral preprocessing methods were investigated before linear discriminant analysis (LDA) or partial least squares (PLS) regression analysis. In particular, the spectra were firstly treated by multiplicative scatter correction (MSC), then by de-trending and, finally, smoothed by a 15-point Savitzky–Golay (2nd derivative, 2nd polynomial order) function [40]. Prior to proceeding to LDA or PLS analysis, a principal components analysis (PCA) was also applied to the 500 wheat samples to detect outliers or any clustering of the data. Sample outliers were detected by using the graphical tools of the Unscrambler^®^ X software, *i.e.*, the Hotelling T² line plot using a critical limit of *p*-value <5% and the influence plot, displaying samples with high leverage. Samples suggested as outliers were removed from the entire set, because their inclusion would have a detrimental effect on the model. This procedure was repeated several times, until a total of 36 samples were considered as outliers, and the remaining 464 samples were used for LDA and PLS model development.

### 3.6. Development and Validation of DON Quantitative Models

Quantitative calibration models were developed using partial least squares (PLS) regression algorithms by correlating the DON results from HPLC reference measurements with the FT-NIR spectra results. The PLS is a spectral decomposition technique, which finds the most relevant factors to explain the variance in the dataset and compares the covariance between the spectral data and DON concentration. Two PLS models were developed (PLS I and II). PLS I was developed on 464 samples in the range of concentrations of ≤50 to 16,000 µg/kg DON, whereas PLS II was developed on 408 samples in the range of concentrations of ≤50 to 6,000 µg/kg DON. In both cases, sample spectra were randomly divided into a calibration set (50% of samples) and a validation set (50% samples) for developing and testing the model, respectively. Performance of the PLS models was evaluated by calculating the coefficient of determination (*r^2^*), the slope of the calibration regression curve and the root mean squared error of calibration (RMSEC). The final PLS models were validated using an independent validation dataset, and the performance was evaluated according to the slope and coefficient of determination of the validation regression curve (*r^2^*) and the root mean standard error of prediction (RMSEP). The models’ accuracy ability was categorized as follows: *r^2^* = 0.50–0.64, usable for rough screening; *r^2^* = 0.66–0.81, usable for screening and some “approximate” calibrations; *r^2^* = 0.83–0.90, usable with caution; *r^2^* = 0.92–0.96, usable in most applications; *r^2^* > 0.98, usable in any applications [41]. The prediction accuracy of models was also evaluated based on the residual predictive deviation (RPD), defined as the ratio of the standard deviation of the reference DON values in the validation set to the RMSEP [42]. The RPD evaluates how well the developed model can predict the DON in the validation set, and the higher is its value, the greater is the prediction ability of the model. Values of RPD from 3.1 to 4.9 are considered fair, and the model is recommended for very rough screening purposes; values from 5.0 to 6.4 indicate that the model is good for quality control; values from 6.5 to 8 indicate that the model is very good for process control; values higher than 8.1 indicate that the model is excellent and usable for any application [43]. Finally, the range error ratio (RER), calculated by dividing the range of the reference DON values in the validation set by the RMSEP, was used as a useful indicator to assess the practical utility of the calibration as a predictive model. Both the RER and RPD standardize the RMSEP value of the model against the range and standard deviation, respectively, of the reference data in the validation set.

### 3.7. Development and Validation of DON Classification Models

In order to develop a chemometric calibration model for the classification of DON-contaminated samples, a linear discriminant analysis (LDA) was applied to the preprocessed spectral measurements. The LDA was carried out assuming equal prior probability for each class; moreover, the first 10 principal components (PCs) of the spectra were used as the explanatory variables to evaluate how wheat classes were separated from each other in the spectral space. Two different approaches were followed to develop LDA models. With the first approach (LDA I), three different classes of samples with different DON content as determined by HPLC analysis were defined: samples with DON content less than or equal to 1,000 µg/kg (Class A); samples with DON content ranging from 1,000 to 2,500 µg/kg (Class B); samples with DON content higher than 2,500 µg/kg (Class C) (LDA I). The DON contamination range for Class B was established by adding and subtracting an arbitrary measurement uncertainty of 750 µg/kg with respect to the EC ML for DON in unprocessed durum wheat (*i.e.*, 1,750 µg/kg). These three classes were chosen to represent three different conditions in terms of DON contamination: compliant samples with DON levels lower than EU ML (Class A), samples with DON concentration to be confirmed by HPLC analysis (Class B) and rejectable (or not compliant) samples with high DON levels (Class C). With the second approach, wheat samples were distinguished into two groups using a cut-off limit to classify them. In particular, three classification models (LDA Models II–IV) were developed by using three different cut-off levels of DON, *i.e.*, 1,000 µg/kg, 1,200 µg/kg and 1,400 µg/kg. Compliant samples were considered those with DON concentrations ≤ the cut-off level (Class A), whereas rejectable (or non-compliant) samples to be confirmed by HPLC analysis were considered those with DON concentrations > the cut-off level (Class B). For each classification model, a total of 464 samples spectra were randomly divided into calibration and validation sets by randomly subdividing the available spectral data into two equal sets (*i.e.*, 50% calibration and 50% validation) (Table 3). The two sets covered the same concentration range of DON.

**Table 3:** Number of durum wheat samples in Classes A, B and C used for Model I and in Classes A and B used for Models II–IV. For each model, the number of samples in the calibration and validation sets was the same.

Model	Number of samples (DON, µg/kg)		
	Class A	Class B	Class C
I	118 (≤1,000)	37 (1,000–2,500)	77 (>2,500)
II	113 (≤1,000)	119 (>1,000)	-
III	118 (≤1,200)	114 (>1,200)	-
IV	123 (≤1,400)	109 (>1,400)	-

The LDA used pooled covariance matrices in calculating the Mahalanobis squared distance [44]. In this algorithm, a sample is assigned into one of the DON concentration groups when the squared distance between the sample and the group is minimized. The Mahalanobis squared distances were then calculated to classify an unknown sample from the validation data set into the DON group with the lowest corresponding distance. The prediction ability of the chemometric models was expressed in terms of overall discrimination rate, false compliant rate and false non-compliant rate, according to the following equations:

(1) $$\text{Overall discrimination rate }(\%)\;=\;\frac{\text{TC}+\text{TNC}}{\text{Ntot}}\;\times \;100,$$

(2) $$\text{False compliant rate }(\%)\;=\;\frac{\text{FC}}{\text{Ntot}}\;\times 100,$$

(3) $$\text{False},\text{ not compliant rate }(\%)\;=\;\frac{\text{FNC}}{\text{Ntot}}\;\times \;100$$

where TC, TNC, FC and FNC denote the number of true compliant, true, not compliant, false compliant and false, not compliant, respectively. N_tot_ denotes the number of samples in the considered class and in the entire set (calibration or validation), respectively. The best classification model was determined as the one with the highest overall discrimination rate and lowest false compliant samples.

The repeatability of the classification models was estimated by analyzing by FT-NIR spectroscopy the laboratory QC sample of ground unprocessed durum wheat. The sample was analyzed ten times under repeatability conditions, and DON levels were predicted by each LDA model (I–IV). For each model, the repeatability was calculated as the coefficient of variation (CV) of the Mahalanobis squared distances in the PCA of the ten measurements. The within-laboratory reproducibility of the classification models was estimated by analyzing the same quality control sample ten times on three different days under within-laboratory reproducibility conditions. For each model the CV was calculated by considering the 30 measurements.

## 4. Conclusions

Classification and calibration methods using Fourier-transform near-infrared (FT-NIR) spectroscopy have been developed to predict DON levels in unprocessed durum wheat samples. The quantitative PLS models showed a very poor classification ability and were not recommended for any purpose. On the other hand, the classification models showed good predictive performance with high overall classification rates and low misclassification of wheat samples and fulfilled the requirement of the European official guidelines for screening methods when a cut-off value of 1,400 µg/kg of deoxynivalenol was used.

The proposed classification model may have practical applications for screening durum wheat samples for deoxynivalenol content, making FT-NIR spectroscopy a powerful and robust tool for monitoring safety programs. Moreover, the speed of the procedure allows the analysis of a large number of samples and reduces the number of samples to be confirmed with a reference method to verify the compliance with regulation.

## References

[1] Canady R.A., Coker R.D., Egan S.K., Krska R., Kuiper-Goodman T., Olsen M., Pestka J., Resnik S., Schlatter J. (2001). Deoxynivalenol. Safety Evaluation of Certain Mycotoxins in Food.

[2] Shephard G.S. (2011). *Fusarium* mycotoxins and human health. Plant Breed. Seed Sci..

[3] Arunachalam C., Doohan F.M. (2013). Trichothecene toxicity in eukaryotes: Cellular and molecular mechanisms in plants and animals. Toxicol. Lett..

[4] Antonissen G., Martel A., Pasmans F., Ducatelle R., Verbrugghe E., Vandenbroucke V., Li S., Haesebrouck F., van Immerseel F., Croubels S. (2014). The impact of *Fusarium* mycotoxins on human and animal host susceptibility to infectious diseases. Toxins.

[5] Pinton P., Oswald I.P. (2014). Effect of deoxynivalenol and other Type B trichothecenes on the intestine: A review. Toxins.

[6] Maresca M. (2013). From the gut to the brain: Journey and pathophysiological effects of the food-associated trichothecene mycotoxin deoxynivalenol. Toxins.

[7] European Commission (2007). Commission Regulation (EC) No. 1126/2007 of 28 September 2007 amending Regulation No. 1881/2006 Setting maximum levels for certain contaminants in foodstuffs as regards Fusarium toxins in maize and maize products. Off. J. Eur. Union.

[8] Ran R., Wang W., Han Z., Wu A., Zhang D., Shi J. (2013). Determination of deoxynivalenol (DON) and its derivatives: Current status of analytical methods. Food Control.

[9] Meneely J.P., Ricci F., van Egmond H.P., Elliott C.T. (2011). Current methods of analysis for thedetermination of trichothecenemycotoxins in food. Trends Anal. Chem..

[10] Lattanzio V.M.T., Pascale M., Visconti A. (2011). Current analytical methods for trichothecene mycotoxins in cereals. Trends Anal. Chem..

[11] Lippolis V., Maragos C. (2014). Fluorescence polarization immunoassays for rapid, accurate and sensitive determination of mycotoxins. World Mycotoxin J..

[12] Li Y., Liu X., Lin Z. (2012). Recent developments and applications of surface plasmon resonance biosensors for the detection of mycotoxins in foodstuffs. Food Chem..

[13] Lippolis V., Pascale M., Cervellieri S., Damascelli A., Visconti A. (2014). Screening of deoxynivalenol contamination in durum wheat by MOS-based electronic nose and identification of the relevant pattern of volatile compounds. Food Control.

[14] McClure W. (2003). Review: 204 years of near infrared technology: 1800–2003. J. Near Infrared Spectrosc..

[15] Santos C., Frafa M.E., Kozakiewicz Z., Lima N. (2010). Fourier transform infrared as a powerful technique for the identification and characterization of filamentous fungi and yeasts. Res. Microb..

[16] Pettersson H., Aberg L. (2003). Near infrared spectroscopy for determination of mycotoxins in cereals. Food Control.

[17] Kos G., Krska R., Lohninger H., Griffiths P.R. (2004). A comparative study of mid-infrared diffuse reflection (DR) and attenuated total reflection (ATR) spectroscopy for the detection of fungal infection on RWA2-corn. Anal. Bioanal. Chem..

[18] Abramovic B., Jajic I., Abramovic B., Cosic J., Juric V. (2007). Detection of deoxynivalenol in wheat by Fourier transform infrared spectroscopy. Acta Chim. Slov..

[19] De Girolamo A., Lippolis V., Nordkvist E., Visconti A. (2009). Rapid and non-invasive analysis of deoxynivalenol in durum and common wheat by Fourier-Transform Near Infrared (FT-NIR) spectroscopy. Food Addit. Contam. Part A Chem. Anal. Control Expo. Risk Assess..

[20] Siuda R., Balcerowska G., Kupcewicz B., Lenc L. (2008). A modified approach to evaluation of DON content in scab-damaged ground wheat by use of diffuse reflectance spectroscopy. Food Anal. Methods.

[21] Bolduan C., Montes J.M., Dhillon B.S., Mirdita V., Melchinger A.E. (2009). Determination of mycotoxin concentration by ELISA and near-infrared spectroscopy in *Fusarium*-inoculated maize. Cereal Res. Commun..

[22] Beyer M., Pogoda F., Ronellenfitsch F.K., Hoffmann L., Udelhoven T. (2010). Estimating deoxynivalenol contents of wheat samples containing different levels of *Fusarium*-damaged kernels by diffuse reflectance spectrometry and partial least square regression. Int. J. Food Microb..

[23] Dvořáček V., Prohasková A., Chrpová J., Štočková L. (2012). Near infrared spectroscopy for deoxynivalenol content estimation in intact wheat grain. Plant Soil Environ..

[24] Czechlowski M., Laskowska M. (2013). The development and validation of the calibration model for the VIS-NIR spectrometer used for the evaluation of deoxynivalenol content in wheat grain directly during combine harvest. J. Res. Appl. Agric. Eng..

[25] Galvis-Sánchez A.C., Barros A.S., Delgadillo I. (2008). Method for analysis dried vine fruits contaminated with ochratoxin A. Anal. Chim. Acta.

[26] Bozza A., Tralamazza S.M., Rodriguez J.I., Scholz M.B.S., Reynaud D.T., Dalzoto P.R., Pimentel I.C. (2013). Potential of Fourier Transform infrared Spectroscopy (FT-IR) to detection and quantification of ochratoxin A: A comparison between reflectance and transmittance techniques. Int. J. Pharm. Chem. Biol. Sci..

[27] Berardo N., Pisacane V., Battilani P., Scandolara A., Pietri A., Marocco A. (2005). Rapid detection of kernel rots and mycotoxins in maize by near-infrared reflectance spectroscopy. J. Agric. Food Chem..

[28] Gaspardo B., del Zotto S., Torelli E., Cividino S.R., Firrao G., Della Riccia G., Stefanon B. (2012). A rapid method for detection of fumonisins B1 and B2 in corn meal using Fourier transform near infrared (FT-NIR) spectroscopy implemented with integrating sphere. Food Chem..

[29] Della Riccia G., del Zotto S. (2013). A multivariate regression model for detection of fumonisins content in maize from near infrared spectra. Food Chem..

[30] Hernández-Hierro J.M., García-Villanova R.J., Gonzáles-Martín I. (2008). Potential of near infrared spectroscopy for the analysis of mycotoxins applied to naturally contaminated red paprika found in the Spanish market. Anal. Chim. Acta.

[31] Fernández-Ibañez V., Soldado A., Martínez-Fernández A., de la Roza-Delgado B. (2009). Application of near infrared spectroscopy for rapid detection of aflatoxin B_1_ in maize and barley as analytical quality assessment. Food Chem..

[32] Tekle S., Bjørnstad A., Skinnes H., Dong Y., Segtnan V.H. (2013). Estimating deoxynivalenol content of ground oats using VIS-NIR spectroscopy. Cereal Chem. J..

[33] Ruan R., Li Y., Lin X., Chen P. (2002). Non-destructive determination of deoxynivalenol levels in barley using near-infrared spectroscopy. Appl. Eng. Agric..

[34] Peiris K.H.S., Dong Y., Bockus B.B., Dowell F.E. Estimation of bulk deoxynivalenol and moisture content of wheat grain samples by FT-NIR spectroscopy. Proceedings of the 2013 ASABE Annual International Meeting.

[35] Garon D., el Kaddoumi A., Carayon A., Amiel C. (2010). FT-IR spectroscopy for rapid differentiation of *Aspergillus flavus*, *Aspergillus fumigatus*, *Aspergillus parasiticus* and characterization of aflatoxigenic isolates collected from agricultural environments. Mycopathologia.

[36] Dachoupakan Sirisomboon C., Putthang R., Sirisomboon P. (2013). Application of near infrared spectroscopy to detect aflatoxigenic fungal contamination in rice. Food Control.

[37] Levasseur-Garcia C., Kleiber D., Surel O. (2013). Infrared spectroscopy used as a decision-making support for the determination of fungal and mycotoxic risk. Cah. Agric..

[38] European Commission (2002). Commission Decision No 657/2002 of 12 August 2002 implementing Council Directive 96/23/EC concerning the performance of analytical methods and the interpretation of results. Off. J. Eur. Commun..

[39] Visconti A., Haidukowski M., Pascale M., Silvestri M. (2004). Reduction of deoxynivalenol during durum wheat processing and spaghetti cooking. Toxicol. Lett..

[40] Savitzky A., Golay M.J.E. (1964). Smoothing and differentiation of data by simplified least squares procedures. Anal. Chem..

[41] Williams P.C. (2004). Near-infrared technology: Getting the best out of light. A Short Course in the Practical Implementation of Near Infrared Spectroscopy for the User.

[42] Fearn T. (2002). Assessing calibrations: SEP, RPD, RER and R^2^. NIR News.

[43] Williams P.C., Williams P., Norris K. (2001). Implementation of near-infrared technology. Near Infrared Technology in the Agricultural and Food Industries.

[44] Johnson D.E., Johnson D.E. (1998). Discriminant analysis. Applied Multivariate Methods for Data Analysts.

